# Determinants of neurological syndromes caused by varicella zoster virus (VZV)

**DOI:** 10.1007/s13365-020-00857-w

**Published:** 2020-06-03

**Authors:** Peter GE Kennedy, Trine H Mogensen

**Affiliations:** 1 Institute of Infection, Immunity and Inflammation, College of Medical, Veterinary and Life Sciences, University of Glasgow, Glasgow, Scotland UK; 2 Departments of Infectious Diseases and Clinical Medicine, Aarhus University Hospital, Aarhus, Denmark; 3 Department of Biomedicine, Aarhus University, Aarhus, Denmark

**Keywords:** Varicella zoster virus, Nervous system, Varicella, Herpes zoster, Encephalitis, POL III, Host genetics, Immunodeficiency

## Abstract

Varicella zoster virus (VZV) is a pathogenic human herpes virus which causes varicella as a primary infection, following which it becomes latent in peripheral autonomic, sensory, and cranial nerve ganglionic neurons from where it may reactivate after decades to cause herpes zoster. VZV reactivation may also cause a wide spectrum of neurological syndromes, in particular, acute encephalitis and vasculopathy. While there is potentially a large number of coding viral mutations that might predispose certain individuals to VZV infections, in practice, a variety of host factors are the main determinants of VZV infection, both disseminated and specifically affecting the nervous system. Host factors include increasing age with diminished cell-mediated immunity to VZV, several primary immunodeficiency syndromes, secondary immunodeficiency syndromes, and drug-induced immunosuppression. In some cases, the molecular immunological basis underlying the increased risk of VZV infections has been defined, in particular, the role of POL III mutations, but in other cases, the mechanisms have yet to be determined. The role of immunization in immunosuppressed individuals as well as its possible efficacy in preventing both generalized and CNS-specific infections will require further investigation to clarify in such patients.

## Introduction

**Fig. 1:**
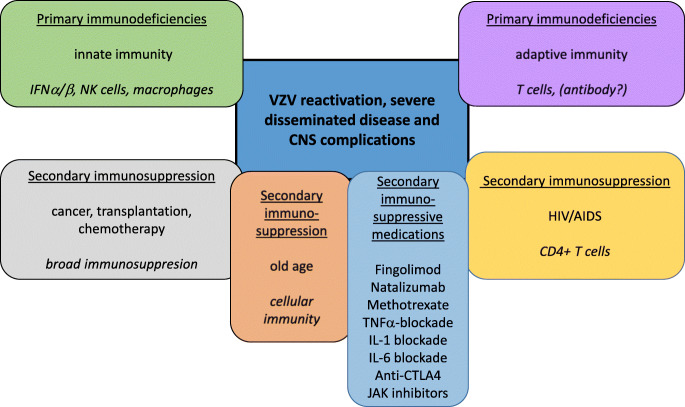
Host factors predisposing to VZV reactivation, severe disseminated disease, and CNS complications. Predisposing factors include primary genetically determined immunodeficiencies in innate or adaptive immunity as well as states of secondary immunodeficiency imparted by cancer, transplantation, chemotherapy, HIV/AIDS, immunosuppressive medications for various autoimmune conditions, age-related immune decline, and others

Varicella zoster virus (VZV) is a pathogenic and ubiquitous human alpha herpes virus which causes varicella (chickenpox) as a primary infection, usually, though not always, in children following which it may become latent in neurons in peripheral autonomic, sensory, and cranial nerve ganglionic neurons along the entire human neuroaxis (Kennedy et al. 1998; Gershon et al. 2015). After decades, either spontaneously or following various triggering factors, VZV may reactivate to cause herpes zoster (shingles) which is a painful vesicular skin eruption in the distribution of a particular dermatome (Nagel and Gilden 2007). Both varicella and herpes zoster may be more frequent and serious in individuals who are immunosuppressed either from diseases disrupting the immune system or because of drug therapy for cancer or various autoimmune conditions (Fig. 1). A variety of neurological conditions may follow both varicella and herpes zoster, and the key determinants for these are now being determined. There is no doubt that host immune factors are of primary importance in the development of these complications, but it also seems reasonable to assume that both viral and host factors may play a role and could also interact.

In this review of the subject, we first provide a brief overview of the neurological syndromes associated with varicella and zoster and then consider the potential viral and host determinants for neurological disease development, focussing on recent evidence implicating the key role of host genetic mutations in increasing susceptibility to VZV-induced nervous system disease.

## Overview of neurological syndromes associated with primary and reactivated VZV

The neurological complications of both varicella and zoster have been described in considerable detail elsewhere (Gershon et al. 2015; Kennedy and Gershon 2018) and will be briefly outlined here. Though varicella is usually an uncomplicated but unpleasant exanthema with a pruritic vesicular rash, it can be a very serious illness in children who are immunocompromised due to illness or drug therapy as well as in the few adults who contract the illness (Kennedy and Gershon 2018). While superadded bacterial infection of the skin, septicaemia, and pneumonia may complicate varicella, the most frequent neurological complication of varicella is encephalitis which refers to severe inflammation of the brain parenchyma (Chaudhuri and Kennedy 2002). This is a rare complication, probably occurring in less than 0.1% of cases though encephalitis itself accounts for about 90% of neurological complications (Kennedy 1987). In some cases, there is a syndrome of acute cerebellar ataxia due to a postinfectious meningoencephalitis that is typical of VZV (Chaudhuri and Kennedy 2002) and accounts for about 50% of all the neurological complications (Kennedy 1987). Much rarer neurological syndromes have also been described in the literature and include myelitis, Reye’s syndrome, congenital varicella syndrome and, very rarely, the Guillain-Barre syndrome (Johnson and Milbourn 1970; Kennedy 1987).

The neurological features of VZV reactivation, usually manifest as herpes zoster, are protean and the observed range of these manifestations is progressively increasing as the viral detection methods for VZV in human tissues become more advanced and rigorously applied in inflammatory nervous system conditions of uncertain cause (Nagel and Gilden 2007). Of all the various complications of zoster, post herpetic neuralgia (PHN) is the most recognized. Both zoster and PHN are increasingly common in the elderly and also the immunosuppressed, probably because of diminished cell-mediated immunity to the virus (Hope-Simpson 1965; Gershon et al. 2015). While zoster produces a painful pruritic dermatomally distributed rash, PHN occurs when the severe dermatomal pain persists after 3 months following healing of the zoster rash (Kennedy et al. 2013). PHN occurs in about 40% of zoster patients over 60 years (Gilden et al. 2000) and is extremely refractory to treatment, and most of which are usually ineffective in relieving the persistent pain. The cause of PHN is not understood though both host and viral factors may be involved (Kennedy et al. 2013; Gershon et al. 2015). Reported risk factors for the development of PHN are an age of more than 50 years, female gender, a zoster viremia detectable by the polymerase chain reaction (PCR) (which currently remains somewhat uncertain), severe or disseminated rash, and the presence of a prodrome and severe pain at the time of its presentation (Wareham and Breuer 2007).

Herpes zoster can rarely be followed by a meningoencephalitis which may run a relatively benign course, and in about 0.25% of zoster cases, an encephalomyelitis may occur (Kennedy 1987). While there is some controversy as to whether this is a separate disease entity from a vasculitis (see below), the authors consider these two conditions as separate. The factors that may increase the duration of this condition include immunosuppression, disseminated zoster lesions, and the presence of extracranial signs (Jemsek et al. 1983).

A VZV-induced vasculopathy is now well characterized and is one of the most important complications of zoster. The spectrum of this vasculopathy has been extended considerably and includes temporal artery involvement (see below), ischemic and hemorrhagic stroke, arterial dissection, transient ischemic attacks, ischemic cranial neuropathies, spinal cord infarction, and cerebral venous thrombosis (Nagel and Gilden 2014; Kennedy 2016). Confusion, seizures, a variety of focal neurological signs, and progressive cognitive decline are typical clinical features of a VZV vasculopathy, and the diagnosis of which may be established by analysis of the cerebrospinal fluid (CSF) and arteriography or MRI angiography (Gilden et al. 2000; Kennedy 2016). Interestingly, the presence and level of anti-VZV IgG antibody in the CSF has been shown to be even more sensitive in diagnosing a VZV vasculopathy than PCR which detects VZV DNA (Nagel et al. 2007). It should be appreciated, however, that there is some uncertainty as to the actual specificity of the CSF antibody/serum antibody ratio as used in some studies for the diagnosis of VZV-related disease. Treatment has been formulated on a relatively small number of cases but usually consists of a 14-day course of intravenous acyclovir and a short 5–7-day course of oral corticosteroids (Kennedy 2016). As is the case for many of the VZV-associated neurological syndromes, however, there is just not enough clinical data available to be certain of the optimal therapeutic regime.

Segmental motor weakness affecting the limbs, intercostal or diaphragmatic muscles may occur in association with zoster infection, and the region of the weakness does not necessarily correlate with the distribution of the zosteriform rash (Kennedy 1987). The prognosis is thought to be generally good. In addition, zoster may also cause a myelitis with characteristic clinical features. Magnetic resonance imaging (MRI) of the relevant region of the spinal cord may help confirm the diagnosis.

It is now recognized that zoster can occur in the absence of the typical rash and this has been termed zoster sine herpete (ZSH) (Gilden et al. 1994). The spectrum of neurological syndromes that have now been described in ZSH has steadily increased to the extent that this diagnosis should be considered in any patient who presents with a presentation of acute, subacute, or chronic brain or spinal cord disease of unknown cause, together with a CSF pleocytosis (Nagel and Gilden 2007, Kennedy 2011). The diagnosis, once suspected, can be established using PCR to detect VZV DNA in the CSF or else serologically by the presence of anti-VZV IgG in the CSF.

Although VZV has been reported to be the likely cause of a high proportion of cases of giant cell arteritis (GCA), this finding remains controversial and is the subject of ongoing investigation. Thus, Gilden et al. (2015) first reported that three quarters of temporal arteries (TAs) from patients who had positive inflammatory pathology contained VZV antigen, mainly in so-called skip regions, and this contrasted with normal TAs of which only 8% contained VZV antigen. Their subsequent study reported that VZV antigen was also present in 64% of TAs that were from patients with suspected GCA but who had negative biopsy pathology as compared with only 22% of normal TAs (Nagel et al. 2015). It is possible that there exists a subset of GCA patients in whom VZV may be playing a causative role, a notion that has clear therapeutic implications, but these findings have not been replicated (Kennedy et al. 2003, Buckingham et al (2018), Ostrowski et al. 2019). Further studies of this phenomenon are ongoing, and the results of which are awaited with interest.

Since zoster frequently affects the first division of the trigeminal nerve, VZV may also cause various types of eye disease, both neurological and primarily ophthalmological. Thus, zoster may cause oculomotor nerve and optic nerve lesions (Kennedy 1987) as well as such local conditions as a keratitis, acute retinal necrosis, a characteristic progressive outer retinal necrosis, retinal hemorrhages, and macular lesions (Gershon et al. 2015). Further, the ability of zoster to cause cranial neuropathies includes a seventh (facial) nerve palsy in association with geniculate zoster, the Ramsay Hunt syndrome, a painful condition that requires treatment with antiviral drugs (Kennedy 2016). In fact, most herpes zoster in adults probably warrants treatment with antiviral drugs.

Finally, very rare cases have been described, in which VZV encephalitis triggers autoimmune NMDA receptor immunoreactivity/encephalitis, possibly through molecular mimicry or “altered self” or as a paraneoplastic phenomenon (Schabitz et al. 2014), although this is more prominent post herpes simplex encephalitis (Armangue et al. 2015).

## Possible viral determinants of neurological disease

Although host factors are probably predominant in determining susceptibility to VZV and its complications, it is still possible that certain viral factors may also play a role in this process. In this context, a critical factor is clearly the number and nature of any coding mutations that may occur in the normal VZV genome. While in theory, any of the ~ 125,000 bases in VZV could mutate and produce viable virus, and in practice, it is more relevant that there may be as many as 300 single nucleotide polymorphisms (SNPs) when comparing two VZV from different geographic clades (D.Depledge personal communication). Whether any detected SNPs confer important functionality in the VZV is certainly possible though not yet known for certain. But these possibilities do provide a feasible background for different VZV strains producing different clinical phenotypes in its human host. Support for this notion is suggested by finding mutations in the vaccine strain VZV that are more likely to cause complications of vaccination.

It is possible that a particularly virulent strain of VZV may exert a clinically significant phenotype, and a recent study provided evidence that this may well be the case. When 11 VZV isolates from patients who had developed PHN were compared with 9 VZV isolates from non-PHN patients for their ability to produce different electrophysiological effects in the ND7/23-Nav1.8 neuroblastoma cell line, it was found that the PHN-associated viruses significantly increased sodium current amplitude in the cell line when compared with non-PHN VZV, wild-type (Dumas), or vaccine VZV strains (Kennedy et al. 2013). These PHN-associated VZV sodium current increases were found to be mediated in part by the Nav 1.6 and Nav 1.7 sodium ion channels. While extrapolation of such in vitro findings to the human scenario should be viewed with some caution, nevertheless this does suggest a possible mechanism of virally induced pain since alterations of sodium channel currents are known to be associated with the sensation of pain (Wood et al. 2004; Garry et al. 2005). In order to gain a greater understanding of the possible role of the virus in determining the various neurological syndromes, it will be important to confirm and extend this type of study and to analyze potential coding mutations in the VZV genome in further detail. Also, it is entirely reasonable to assume that both host and viral factors may interact to influence the development of the various VZV clinical syndromes.

## Host factors that determine susceptibility to VZV-associated neurological disease

### Primary immunodeficiencies predisposing to severe Varicella, Zoster and VZV CNS infection

**Fig. 2:**
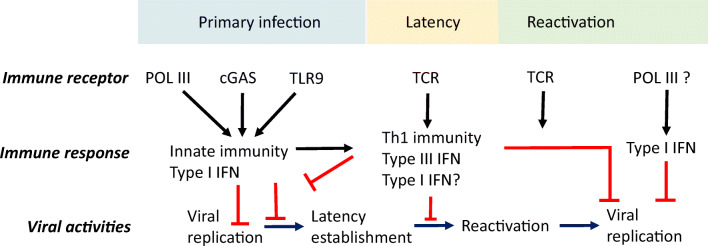
Antiviral immune responses to VZV during different phases of infection. Roles for innate and adaptive immune receptors and responses in primary VZV infection, latency, and reactivation. Innate immune responses include recognition of VZV DNA by cytosolic DNA sensors POL III and cGAS, as well as by endosomal TLR9 to generate type I interferon (IFNα/β) and prime adaptive immunity. NK cells also play a role through cytotoxic activities. Later, adaptive immune responses mediated by T cells produce type II IFN (IFNγ) and other cytokines. A combination of cellular immunity and IFNs is suggested to be involved in maintaining latency and preventing viral replication and reactivation. POL III, RNA polymerase III, cGAS cytosolic GMP-AMP synthase, TLR, Toll-like receptor

The precise immunological correlates of protective immunity to VZV remain incompletely understood. This is partly because VZV is a strict human pathogen and only limited in vivo data from (humanized) mouse models or post-mortem examination of human trigeminal ganglia are available. In this context, the study of human inborn errors of immunity conferring increased susceptibility to VZV has proven particularly valuable. It is clear that a prominent role is played by cellular immunity exerted by T cells and natural killer (NK) cells (Duncan and Hambleton 2015; Gershon et al. 2015; Zerboni et al. 2014). The picture is less clear in the case of the role of humoral immunity, and antibodies are generally not believed to contribute to a large extent, as also suggested by the absence of VZV infection as a prominent clinical phenomenon in human antibody deficiencies. All types of interferons (IFNs) serve important functions in restricting VZV replication and spread, particularly during the viremic phase and in the skin during varicella, and also possibly in maintaining latency in sensory neuronal ganglia (Carter-Timofte et al. 2018b; Duncan and Hambleton 2015) (Fig. 2). It is therefore relevant that recent studies demonstrated an antiviral role of both IFNα/β and IFNγ *in vitro* (Sen et al. 2018).

Several primary immunodeficiencies (PIDs) have been shown to predispose to severe disseminated primary varicella, frequent and extensive zoster, varicella pneumonia, and in some cases CNS complications (for an overview, see Table 1). The classical example is severe combined immunodeficiency (SCID) with defects in T cells, B cells, and NK cells caused by defects in IL2RG, RAG1/2, ADA, JAK3, and IL7R genes, in which an increased risk of disseminated VZV infection has been well-recognized for many years (Arvin et al. 2010; Carter-Timofte et al. 2018b; Fischer et al. 2015; Zerboni et al. 2014). Other combined PIDs mainly affecting T cells, NK cells, and to a lesser extent B cells, including CORONIN1A (Yee et al. 2016), Wiskott Aldrich syndrome (Albert et al. 2011), CARMIL2 (Schober et al. 2017), MAGT1 (Ravell et al. 2020), STK4 (Abdollahpour et al. 2012), and CXCR4 (Heusinkveld et al. 2017) deficiencies, have also been associated with recurrent zoster and/or persistent skin infection (Al-Herz and Essa 2019). The central role exerted by T cells in anti-VZV immunity is further demonstrated by the occurrence of severe varicella, pneumonia, or chronic VZV infection described in conditions involving the T cell surface molecules CD27 (Alkhairy et al. 2015) and CD70 (Abolhassani et al. 2017) as well STIM1 (Picard et al. 2009). Recently, a defect in DNA polymerase delta1 causing a combined immunodeficiency particularly affecting naïve T cells was described in three children with recurrent infections, including one with encephalitis during primary varicella (Cui et al. 2020). Hemorrhagic varicella, zoster, and keratitis were documented in STAT5B deficiency (Bezrodnik et al. 2015) and DOCK2 deficiency affecting T cells and NK cells with impaired IFNα responses (Dobbs et al. 2015). Of particular interest is the description of a patient with vaccine strain VZV-induced CNS vasculopathy as the presenting feature of DOCK8 deficiency (Sabry et al. 2014; Zhang et al. 2009). Severe disseminated infection with herpesviruses, including VZV, herpes simplex virus (HSV), and cytomegalovirus (CMV), was originally described in a patient with NK cell deficiency, many years later realized to be caused by a genetic variant in the myeloid transcription factor GATA2 causing MonoMAC and also involving cytopenia in monocytic and B cell lineages in addition to NK cell deficiency (Biron et al. 1989; Hsu et al. 2011). Subsequently, reports have described the rare occurrence of hemophagocytic lymphohistiocytosis (HLH) during VZV infection in patients with this disease (Prader et al. 2018; Spinner et al. 2014) as well as in other PIDs distinctive from classical HLH genetic defects (Bode et al. 2012). Moreover, disseminated VZV infection has been observed in other states of impaired NK cell number or function, such as MCM4 (Gineau et al. 2012) and GINS1 deficiencies (Cottineau et al. 2017).

**Table 1:** Primary immunodeficiencies associated with severe VZV infection and/or CNS complications

PID/gene	Defective immune cells	Clinical presentation with VZV disease	References
SCID^a^	T, B, and NK cells	Diss. infection	Fischer et al. (2015)
CORO1A	T and B cells	Diss. infection, pneumonitis, meningitis	Yee et al. (2016)
WAS	All leukocytes	Severe recurrent zoster	Albert et al. (2011)
CARMIL2	T and B cell	Recurrent varicella	Schober et al. (2017)
MAGT1	T and NK cells	Severe varicella, recurrent zoster	Ravell et al. (2020)
STK4	T and B cells	Recurrent severe zoster	Abdollahpour et al. (2012)
CXCR4	T and B cells, neutrophils	Recurrent zoster	Heusinkveld et al. (2017)
CD27	T cells	Severe varicella	Alkhairy et al. (2015)
CD70	T cells	Severe varicella	Abolhassani et al. (2017)
STIM1	T cells	Severe varicella, cellulitis	Picard et al. (2009)
POL D1	T cells	Severe recurrent varicella, encephalitis	Cui et al. (2020)
STAT5B	T and NK cells	Hemorrhagic varicella, zoster, keratitis	Bezrodnik et al. (2015)
DOCK2	T and NK cells	Hemorrhagic varicella, pneumonitis	Dobbs et al. (2015)
DOCK8	T, B, and NK cells	Diss. VZV, CNS vasculopathy	Zhang et al. (2009); Sabry et al. (2014)
GATA2	Monocytes, NK, B cells	Diss. infection, HLH	Biron et al. (1989); Prader et al. (2018)
MCM4	NK cells	Severe varicella	Gineau et al. (2012)
GINS1	NK cells	Severe varicella	Cottineau et al. (2017)
IFNGR	All cells	Varicella pneumonia, CNS infection	Roesler et al. (1999)
STAT1 LOF	All cells	Diss. varicella	Dupuis et al. (2003)
TYK2	All cells	Recurrent zoster	Kreins et al. (2015)
POL III (POLR3 A, C, F)	Leukocytes	Encephalitis, CNS vasculitis, pneumonia	Ogunjimi et al. (2017); Carter-Timofte et al. (2018a)

*PID* primary immunodeficiency; *LOF* loss of function; *HLH* hemophagocytic lymphohistiocytosis

^a^SCID genes: IL2RG, RAG1, RAG2, ADA, JAK3, IL7R

Finally, the group of PIDs that interfere with macrophage function and defense against intracellular pathogens may predispose to infection with mycobacteria and viruses and include defects in the IFNγ receptor (IFNγR) (Roesler et al. 1999), STAT1 loss-of-function (LOF) (Dupuis et al. 2003), and the tyrosine kinase (TYK)2 (Kreins et al. 2015) downstream of IFNα/β and IFNγ receptors.

More recently, the innate cytosolic DNA sensor POL III was added to the list of PIDs predisposing to VZV infection through the identification and characterization of an autosomal dominant inherited loss-of-function POL III defect in four otherwise healthy children with severe VZV in the CNS or lungs (Ogunjimi et al. 2017). RNA polymerase III is a 17-subunit enzyme with dual functions in both promoter dependent transcription of tRNA and rRNA as well as in innate sensing of AT-rich DNA, converting this into 5′-phosphorylated single-stranded RNA, which serves as a ligand for the RNA sensor RIG-I (Ablasser et al. 2009; Chiu et al. 2009). Previously described associations between POL III defects and human disease include the finding of mutations in *POLR3A* and *POLR3B* in the recessive H4 syndrome with severe hypomyelinating leukoencephalopathy (Bernard et al. 2011; Saitsu et al. 2011)*.*The discovery of functional POL III deficiency suggests an important contribution of innate immunity to antiviral defenses against VZV through recognition of the AT-rich VZV genome and demonstrates a major role of type I IFN. A subsequent report described *POLR3F* mutations in monozygotic adult twins both experiencing repeated CNS vasculitis presenting in a stroke-like manner with hemiparesis, sensory deficits, and headache, and clinically diagnosed as recurrent VZV CNS reactivation (Carter-Timofte et al. 2018b). This study, together with another description of POL III defects in adults with VZV encephalitis, suggests that POL III may be important in protection against VZV CNS disease, not only during primary VZV infection but also during reactivation, and thus potentially play a role in controlling VZV latency, although the cellular and molecular correlates of this are not well understood at present (Carter-Timofte et al. 2018a, b, 2019). Whether immunodeficiencies predisposing to VZV encephalitis versus those predisposing to vasculitis are intrinsically different, remains to be studied in detail in larger studies. There is clearly a different pathogenesis between classical VZV meningoencephalitis and the presence of vasculitis developing in some cases of VZV CNS infection (Gilden et al. 2009). At the cellular level, increased viral replication, which for VZV is known to also include endothelial cells within the CNS, may evoke enhanced inflammatory responses secondary to the presence of the virus and thereby explain, at least in part, the development of vasculitis and immunopathology in these patients.

The precise mechanisms whereby POL III restricts VZV in an apparently relatively specific manner have not been fully elucidated. Remaining unanswered questions include (i) issues related to its cell type specificity and redundancy, (ii) the specificity of POL III defects in VZV infection, (iii) its role in primary infection (primarily children) versus reactivation (primarily adults), (iv) its function in innate sensing versus priming of adaptive immunity, and (v) whether Pol III exerts its antiviral role by one (i.e., innate VZV sensing) or whether several different mechanisms may be involved and in which cellular context.

Previous studies have demonstrated high expression levels of POL III in PBMCs, whereas expression of other DNA sensors, such as cGMP-AMP synthase (cGAS), otherwise considered the dominant cytosolic DNA sensor, was relatively low in this cell population (Ogunjimi et al. 2017). Consequently, POL III may play an important role in DNA sensing in PBMCs in particular, partly explaining why POL III defects may be associated with increased susceptibility to infection with pathogens replicating in these hematopoietic cell types. In the case of VZV, both monocytes and T cells are considered important during the viremic phase of infection, and thus defects in innate VZV sensing in these cell types may predispose to high grade viremia and VZV CNS infection in POL III deficiency. In contrast, the adaptor stimulator of IFN genes (STING), acting downstream of DNA recognition through cGAS, was recently reported to participate in antiviral immunity to VZV infection in human dermal fibroblasts and keratinocytes (Kim et al. 2017). Taken together, there are now several studies suggesting that different families of DNA sensors may play individual but dominant roles in VZV infection in different cell types and tissues. Detailed immunological and virological analyses in fibroblasts, endothelial cells, and CNS-resident cells will be needed in order to gain deeper insights into the immunological mechanisms controlling VZV in different anatomical compartments. This issue is particular pertinent in understanding which immune receptors and pathways are responsible for maintaining latency in sensory/autonomic ganglia, and whether POL III plays a role in preventing VZV reactivation.

Regarding the question of specificity, the picture that emerges is that there is some degree of specificity between individual immunodeficiencies and susceptibility to different (herpes)virus infections in the CNS. For instance, it is well established that defects of the TLR3 signaling pathway increase susceptibility to HSV encephalitis (HSE) (Lim et al. 2014; Zhang et al. 2007), and remarkably, POL III mutations were not identified in whole exome sequencing of large cohorts of HSE patients (Lim et al. 2014). On the other hand, impaired POL III function is now known to predispose to VZV encephalitis in both children and adults (Carter-Timofte et al. 2018b). In this setting, the specificity is hypothesized to be due to the essential role of TLR3 in intrinsic immunity to HSV1 in the CNS, whereas POL III, which mainly senses AT-rich DNA, seems to be the dominant sensor of the VZV genome (Ogunjimi et al. 2017). Indeed, it has been suggested that the particular high-AT content of the VZV genome, as opposed to the genomes of HSV and other herpesviruses, may contribute to a certain degree of specificity between POL III defects and increased susceptibility to severe VZV. Overall, the specificity and associations between individual innate sensing pathways and infection by different types of herpesviruses remain to be fully elucidated.

Based on the findings that POL III defect seems to be found in a small fraction of both children in association with primary infection/varicella and adults during reactivation, this raises the question of the role of an innate immune sensor in adaptive immunity. Whereas the available data suggest a key role in VZV DNA sensing and generation of antiviral type I IFN responses, a role for POL III in priming adaptive immune responses is certainly also a possibility. However, to date, no major adaptive immune deficiency in B and T cells has been identified in patients with VZV CNS infection during reactivation from latency (Carter-Timofte et al. 2019, 2018a). However, it is possible that POL III may act by different mechanisms in controlling VZV (viremia) during primary infection in (mainly) children as opposed to secondary infection/reactivation in adults. Maintaining VZV latency in sensory and autonomic ganglia (Kennedy et al. 1998) may be exerted through generation of type I IFN, which has been reported to play a role in this context, or alternatively by other mechanisms in interacting with adaptive immune components, such as T cells (Gershon et al. 2015; Pourchet et al. 2017; Zerboni and Arvin 2015).

It remains an open question as to whether POL III acts exclusively through the generation of type I IFNs after innate sensing of foreign DNA in the cytosol, or whether POL III may also exert antiviral functions through other mechanisms, possibly in the nucleus. The basis for such speculations comes from a recent study demonstrating an antiviral role of a POL III-generated 5S ribosomal RNA pseudogene transcript RNA5SP14 during HSV infection (Chiang et al. 2018). The mechanism was demonstrated to involve release of the otherwise protein bound RNA5SP14 transcript secondary to virus-induced shutdown of cellular protein synthesis, thereby allowing the transcript to relocalise from the nucleus to the cytosol and to stimulate RIG-I-dependent IFNα/β production (Chiang et al. 2018).

Further investigations of the molecular and cellular mechanisms are required to confirm or clarify the role of IFN-mediated immunity in VZV infection. More specifically, it remains to be determined whether POL III is a VZV-specific antiviral immune sensor, possibly related to the particularly AT richness of the VZV genome, and whether it exerts its role in the nucleus or exclusively in the cytoplasm (Carter-Timofte et al. 2018b). In this context, it is also important to clarify the expression of different VZV pattern recognition receptors (PRRs) in different cell types, and the level of redundancy. These factors may provide insights into protective immunity to VZV and whether failing immune mechanisms predisposing to VZV infection in the CNS are failing mainly in the periphery, e.g., during the viremic phases of infection or the latency mechanisms in the sensory ganglia, or, in the brain parenchyma after VZV has gained access into the CNS. A major portion of the defect may well be in clearing tissue infection, since progressive tissue infection is the deleterious outcome. Importantly, it remains unknown how prevalent the POL III defect is in the population. Of clinical importance, answering some of these questions should be relevant to decisions regarding prophylaxis and treatment of VZV CNS infections.

Overall, T cell and NK cell defects tend to cause mainly severe skin and mucous manifestations, whereas defects involving innate IFN signaling are more fulminating and prone to involve CNS complications. Therefore, further cellular and molecular characterization of these PIDs are warranted to clarify the contributions from T, NK, and tissue-resident cells as well as type I and II IFN in the context of VZV infection.

### Secondary immunodeficiencies/immunosuppression in VZV CNS infection

In addition to genetically determined primary immunodeficiencies, a number of other conditions, i.e., secondary immunodeficiencies, encompassing malignancy, transplantation, HIV infection/AIDS, immunosuppressive medication, and age-related immune decline, may also predispose to severe VZV infection, including CNS infection.

#### Cancer and transplantation

Among the first conditions recognized to increase significantly the risk of severe disseminated VZV reactivation were cancer (Schimpff et al. 1972; Sokal and Firat 1965; Wong and Hirsch 1984) and hematopoietic or solid organ transplantation (Champlin and Gale 1984; Luby et al. 1977), mostly ascribed to the significant decline in cellular immunity in these conditions. Moreover, various classes of chemotherapy will invariably increase the risk of VZV complications. For example, treatment of multiple myeloma patients with the proteasome inhibitor bortezomib was associated with a 13% risk of VZV reactivation (Chanan-Khan et al. 2008). Another study specifically addressed the prevalence of severe VZV manifestations, including VZV encephalitis and disseminated VZV, in multiple myeloma patients treated with the thalidomide-derivative lenalidomide and reported such complications in 10 of 93 patients (Konig et al. 2014). Likewise, a substantial number (10 out of 132) patients experienced VZV reactivation within 2 years of stem cell transplantation (Konig et al. 2014). Neurological manifestations of VZV reactivation during treatment of non-solid malignancies have also been described (De Broucker et al. 2012; Rodenburg et al. 2017; Tattevin et al. 2001). This was confirmed by results from the Swiss transplantation cohort recently reporting on severe VZV reactivation in patients following solid organ transplantation (van Delden et al. 2020). Presentation with HLH was described in two adolescents on immunosuppressive therapy for graft-versus-host disease after atypical and severe VZV reactivation (van der Werff ten Bosch et al. 2009). On the other hand, it is important to note that VZV neurological complications have also been documented in a case series in which only half of patients suffered from a known immunodeficiency (Becerra et al. 2013; De Broucker et al. 2012).

#### HIV/AIDS

During the emergence of HIV and AIDS, it became clear that VZV is a cause of CNS infection in HIV-infected individuals with severely impaired CD4 T cell immunity (Burke et al. 1997; Gray et al. 1994; Kennedy 1988; Whitley and Gnann Jr. 1995). In a large study of more than 500 HIV patients with neurological disorders, VZV was diagnosed in the CNS in 2.5%, underscoring that VZV is only one among several other infectious complications in this patient group (Cinque et al. 1996). Based on other studies, VZV reactivation with CNS and ocular manifestations was estimated to occur in up to 11% of HIV positive individuals before the introduction of highly active antiviral therapy (HAART) (Burke et al. 1997). Investigation of CSF samples from HIV positive individuals revealed anti-VZV intrathecal antibody synthesis in 16%, suggesting a significant disease burden of subclinical VZV CNS infection in this patient population (Birlea et al. 2011). Reactivation of VZV in the CNS in AIDS patients is generally associated with a severe diffuse meningoencephalitis, and in some cases, vasculitis, granulomatous angiitis or myelitis (Gilden et al. 2009; Corti et al. 2015; Kennedy 1988). Although myelitis is an extremely rare complication of VZV infection, it is relatively more prevalent in immunocompromised individuals, including AIDS patients, who are prone to atypical presentations and poorer outcomes (Manian et al. 1995). Chronic VZV encephalitis is seen almost exclusively in AIDS patients and manifests with multifocal lesions in the white matter with small vessel vasculitis and demyelinization as well as ischemic and hemorrhagic infarcts (Gnann Jr. 2002; Kleinschmidt-DeMasters et al. 1996). Moreover, several cases of VZV aneurysmal vasculopathy have been reported, and indeed it is possible that many cases of HIV-associated vasculopathy are in reality cases of VZV vasculopathy (Gilden et al. 2009; Tomkins et al. 2018). As an additional complicating factor, VZV-mediated vasculitis presenting as stroke may be caused by underlying CNS immune reconstitution inflammatory syndrome (IRIS) during introduction of antiviral treatment in severely immunocompromised patients (Newsome and Nath 2009; Teo et al. 2014), largely mediated by CD8+ T cells (Venkataramana et al. 2006) and requiring corticosteroids for dampening immune responses and for clinical stabilization. A very rare manifestation of VZV meningitis with CSF cytological changes suggestive of primary CNS lymphoma due to large lymphoid cells has also been described, suggesting caution in the differential diagnosis between viral meningitis and lymphoma, particularly in HIV-infected patients (Park et al. 2003). Although less frequently encountered since the introduction of HAART, ocular manifestation associated with VZV reactivation represents a well-established clinical spectrum occurring more frequently in AIDS patients than in immunocompetent individuals (Batisse et al. 1996; Franco-Paredes et al. 2002; Galindez et al. 1996; Liu et al. 2005). Finally, zoster, particularly when occurring in younger individuals, may be an indicator disease of underlying HIV infection, although it is entirely possible to experience zoster without any obvious immunodeficiency (Melbye et al. 1987; Sogaard et al. 2012).

#### VZV reactivation during treatment with immunomodulatory medications

Secondary immunosuppression through various immunomodulating therapies is a well-established and increasing problem, particularly given that indications for the use of such therapies in rheumatology, gastroenterology, dermatology, neurology, etc. are steadily increasing as are both the spectrum and the availability of such medications. Drugs that interfere with T cell and NK cell immunity or with innate type I IFN responses might be expected to increase the risk of severe VZV, including VZV CNS complications, whereas drugs interfering with humoral immunity are more likely to cause a less severe risk of viral infection.

Natalizumab, an anti-α4β1-integrin antibody inhibiting leukocyte migration across the blood brain barrier into the CNS utilized in the treatment of multiple sclerosis (MS), has been reported to be associated with progressive multifocal leukoencephalopathy (PML) caused by JC virus, and also increases the risk of VZV encephalitis and myelitis (Bourre et al. 2013; Fine et al. 2013; Yeung et al. 2013). The anti-CD52 antibody alemtuzumab approved for the treatment of MS and leukemia, which depletes circulating T and B cells, was also reported to cause severe VZV infection, although not involving the CNS (Williamson and Berger 2015). This drug would be a mild mimic of SCID with the absence of B, T, and NK cells. Another drug used for the treatment of MS is the sphingosine-1-phosphate-receptor-modulator fingolimod, which has also been related to several examples of fulminating cases of disseminated VZV infection, encephalitis, and vasculitis, alone or in combination with other immunomodulating agents (Bourdette and Gilden 2012; Cohen et al. 2010; Issa and Hentati 2015; Ratchford et al. 2012). The mechanism whereby fingolimod predisposes to VZV reactivation is not well understood but has been suggested to stem from the trapping of naïve and memory T cells in lymph nodes causing insufficient T effector cell function to maintain VZV latency (Williamson and Berger 2015).

In addition to the specific examples mentioned above, several studies have reported an increased risk of VZV reactivation in patients with autoimmune conditions receiving various different immunosuppressive medications, including anti-TNFα treatment (Burmester et al. 2014; Strangfeld et al. 2009), including cases of zoster ophthalmicus and meningitis (Serac et al. 2012), glucocorticoids, cyclophosphamide, azathioprine, leflunomide, anti-IL-1, anti-IL-6, and methotrexate (Antonelli et al. 1991; Cacciapaglia et al. 2015; Wolfe et al. 2006), although conflicting results have been obtained for the latter (Zhang et al. 2012). It is, however, clear that the combination of Methotrexate with glucocorticoid is a risk factor for VZV reactivation (McLean-Tooke et al. 2009). While some of these cases were severe, they were characterized by severe cutaneous or disseminated VZV infection rather than infection in the CNS (Cacciapaglia et al. 2015). Among the immune checkpoint inhibitors, severe disseminated VZV was also reported, particular for anti-CTLA4 inhibitors interfering with T cell activation (Del Castillo et al. 2016).

Within the group of more recently introduced immunosuppressive medications, the JAK inhibitors are relevant. These selective inhibitors of Janus kinases act to block JAK-STAT signaling downstream of the type I, II, and III IFN receptors. All JAKs play an important role in immune response and development, with JAK1 and JAK2 also involved in hematopoiesis. JAK1 is required for both type I and type II IFN signaling pathways, whereas JAK3 is essential for lymphocyte development and function (Mogensen 2018). JAK inhibitors are utilized and approved for treating autoinflammatory conditions, in which type I IFN and IFN-stimulated gene (ISG) signatures in blood are prominent, the so-called interferonopathies, such as Aicardi-Goutieres syndrome, STING-associated vasculopathy with onset in infancy (SAVI), STAT1 gain of function, and chronic atypical neutrophilic dermatosis with lipodystrophy and elevated temperature (CANDLE) (Crow 2011; Sanchez et al. 2018). Additionally, JAK inhibitors have been approved for the treatment of rheumatoid arthritis, psoriasis, and inflammatory bowel disease when other treatments fail (Colombel 2018). Interestingly, the major infection risk noticed so far has been VZV reactivation, and this effect appears to be relatively specific for VZV, since no other viral infections were noted (Colombel 2018; Harigai 2019; Marra et al. 2016; Sandborn et al. 2017; Winthrop et al. 2016). Specifically, VZV meningoencephalitis was described in a patient receiving the JAK inhibitor ruxolitinib for myelofibrosis (Eyal et al. 2017). In line with these findings, VZV reactivation/zoster was also reported in studies using anti-IFNα antibodies to treat systemic lupus erythematosus (Furie et al. 2017). These secondary immunodeficiencies thus represent the correlate of the findings in PIDs either interfering with induction of type I IFN responses as seen in POL III and TLR3 deficiencies, or interfering with responses to type I and type II IFN as documented in STAT1 LOF and TYK2 deficiencies and thus underscore the fundamental importance of IFNs in protective immunity against VZV.

Overall, VZV reactivation is more common in patients with rheumatoid arthritis and systemic lupus erythematosus, because of their inherently disturbed immune system (Forbes et al. 2014). Indeed, it should always be remembered that the emergence of opportunistic infection, including VZV reactivation, in individuals with autoimmune and autoinflammatory diseases and treatment with immunomodulatory medications, represents the combined effect of the immunedysregulation imparted by the diseases *per se* together with the immunosuppression exerted by the drug.

#### Other factors predisposing to VZV reactivation

Waning immunity occurring with increasing age is associated with a significantly increased risk of VZV reactivation, usually causing herpes zoster which may be accompanied by CSF pleocytosis and a degree of meningitis or, more rarely, encephalitis (Gilden et al. 2009; Thomas and Hall 2004). This increased susceptibility to VZV reactivation is attributed mainly to a general decrease in cellular immunity, and, possibly more specifically, to reduced levels of protective immunity after primary varicella or VZV vaccination in childhood. Other epidemiological factors influencing the risk of VZV reactivation are recent varicella exposure (protective), time of primary infection/varicella, sex, ethnicity, and possibly psychological and social stress-related factors (Thomas and Hall 2004). For example, a large community-based study reported a strong protective effect associated with recent contacts with varicella patients (Garnett and Grenfell 1992; Thomas et al. 2002), and another study found a trend towards reduced risk of zoster in people born in subtropical/tropical countries with evidence of late-onset varicella (Longfield et al. 1990). Moreover, women appear to be at increased risk, whereas some studies have suggested a lower risk in non-whites, although the specific immunological or genetic correlates of these findings are not entirely clear (Nagasako et al. 2003). However, none of the epidemiological studies cited above specifically addressed the risk of VZV CNS disease in the setting of VZV reactivation in these different populations.

## Conclusion

VZV infection can be largely prevented in the general population, since VZV vaccines that provide effective protection have been developed, including a live attenuated vaccine against varicella and a subunit vaccine for adults and the elderly against herpes zoster. However, a particular problem remains in individuals with severe PID, particularly in cases of profound cellular PID, in whom the live vaccine may pose a threat for serious disseminated VZV infection. More research is required to define more precisely the role of VZV immunization in immunosuppressed individuals. With increased clinical use of a wide variety of immunosuppressive drugs for an increasing number of clinical conditions, it seems very likely that the frequency and range of VZV infections, including those specifically affecting the central and peripheral nervous systems, will be reported. Host immune factors seem to be more important than intrinsic virological factors in predisposing susceptible individuals to VZV infections, but the specific molecular pathways involved in these remain to be elucidated in greater detail.

## Abbreviations

ADA: Adenosine deaminase
CARD11: Caspase recruitment domain containing protein
CARMIL2: Capping protein Arp2/3, myosin linker-I linker
cGAS: Cyclic GMP-AMP synthase
CMV: Cytomegalovirus
CSF: Cerebrospinal fluid
CTLA-4: Cytotoxic T lymphocyte-associated antigen 4
CXCR4: C-X-C Chemokine receptor type 4
DOCK: Dedicator of cytokinesis
GINS1: Go-ichi-ni-san
HAART: Highly active anti-retroviral treatment
HLH: Hemophagocytic lymphohistiocytosis
HSV: Herpes simplex virus
IFN: Interferon
IL7R: Interleukin 7 receptor
IL2RG: Interleukin 2 receptor gamma
IRIS: Immune reconstitution inflammatory syndrome
JAK: Janus kinase
MAGT1: Magnesium transporter 1
MCM4: Minichromosome maintenance complex component 4
MS: Multiple sclerosis
NK: Natural killer
PHN: Post herpetic neuralgia
PID: Primary immunodeficiency
PML: Progressive multifocal leukoencephalopathy
POL III: RNA polymerase III
PRR: Pattern recognition receptor
SCID: Severe combined immunodeficiency
STIM1: Stromal interacting molecule 1
STING: Stimulator of interferon genes
STAT: Signal transducer and activator of transcription
STK4: Serine/threonine protein kinase 4
TYK: Tyrosine kinase
VZV: Varicella zoster virus
